# Supplementary material for: Depleting Mycobacterium tuberculosis of the transcription termination factor Rho causes pervasive transcription and rapid death
Source: Nat Commun. 2017 Mar 28;8:14731. doi: 10.1038/ncomms14731 (PMC5379054; doi:10.1038/ncomms14731)
Supplement: Supplementary Information — Supplementary Figures, Supplementary Tables and Supplementary References [file ncomms14731-s1.pdf]

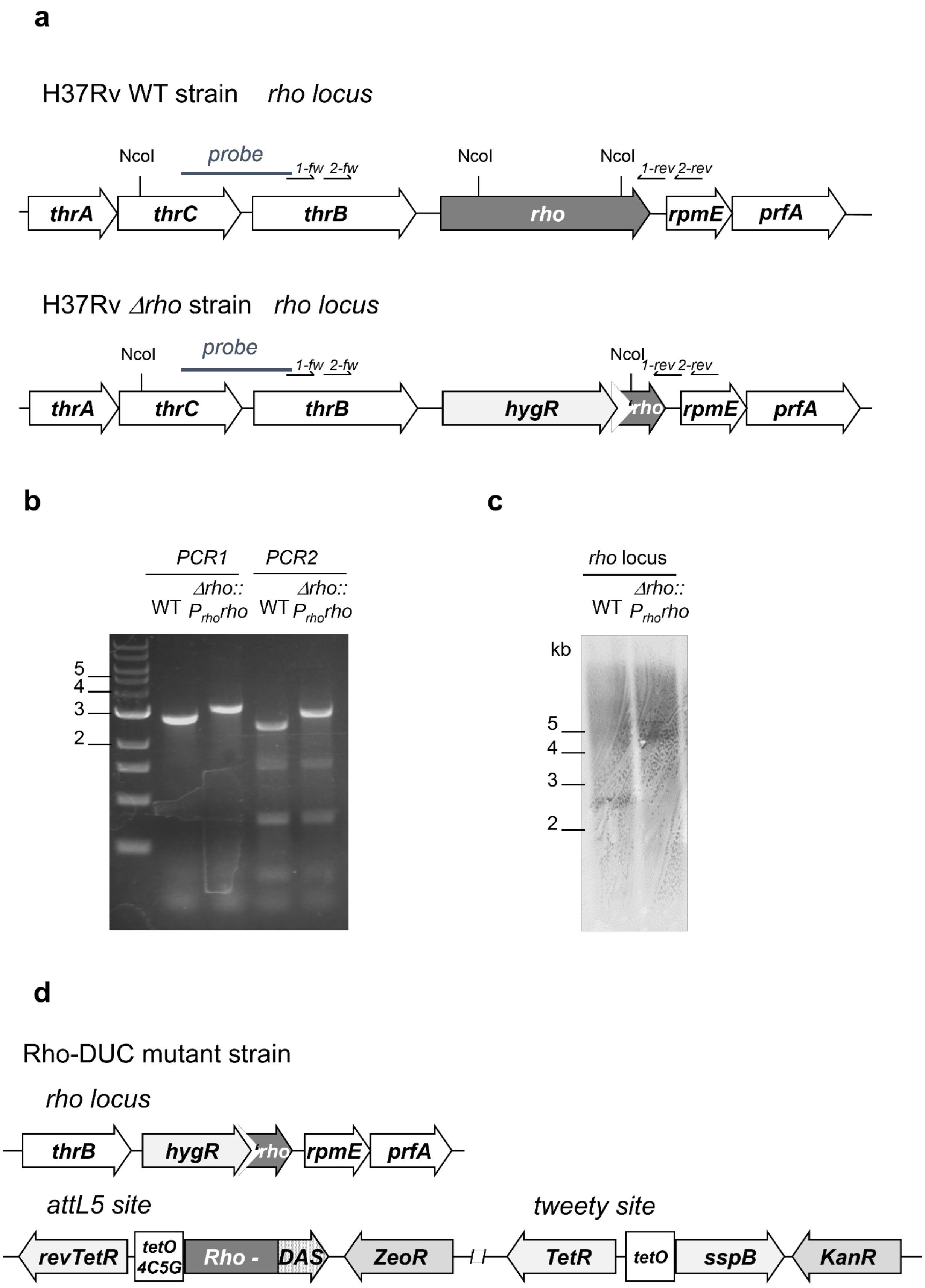
a
H37Rv WT strain rho locus
NcoI
probe
1-fw 2-fw
NcoI
NcoI
1-rev 2-rev
thrA
thrC
thrB
rho
rpmE
prfA
H37Rv Δrho strain rho locus
NcoI
probe
1-fw 2-fw
NcoI
1-rev 2-rev
thrA
thrC
thrB
hygR
'rho
rpmE
prfA
b
PCR1
PCR2
WT
Δrho::Prhorho
WT
Δrho::Prhorho
5
4
3
2
c
rho locus
WT
Δrho::Prhorho
kb
5
4
3
2
d
Rho-DUC mutant strain
rho locus
thrB
hygR
'rho
rpmE
prfA
attL5 site
revTetR
tetO 4C5G
Rho -
DAS
ZeoR
tweety site
TetR
tetO
sspB
KanR

Supplementary Figure 1 │ Construction of *M. tuberculosis* Rho-DUC

(a) Representation of the *rho* locus in the genome *M. tuberculosis* H37Rv wild-type (WT) and *M. tuberculosis Δrho::PrhoTb*, in which the WT copy of *rho* has been replaced with a hygromycin resistance (*hygR*) cassette. The primers used for the discriminative PCRs shown in (b) are indicated as arrows above the genes. The site recognized by the restriction enzyme NcoI as well as the region probe used in the southern blot verification of the strain genotype (c) are indicated as well.

(**b**) Discriminative amplification of the *rho* locus of the WT strain and *Δrho::PrhoTb* using the primers 1-fw and 1-rev or 2-fw and 2-rev for respectively the PCR 1 or 2. The expected sizes of the products are 2795 bp for the WT and 3317 bp for the mutant in the PCR 1 and 2548 bp for the WT and 3070 bp for the mutant in the PCR 2. In addition, the insertion at the correct locus has also been verified by sequencing.

(**c**) *rho* locus specific probing by southern blot of the NcoI digested DNA, of WT and *Δrho::PrhoTb*. Expected sizes of the fragments recognized by the probe are 2377 bp and 4576 bp for the WT and the mutant respectively.

(**d**) Genotype of the Dual-Control *rho* strain (Rho-DUC).

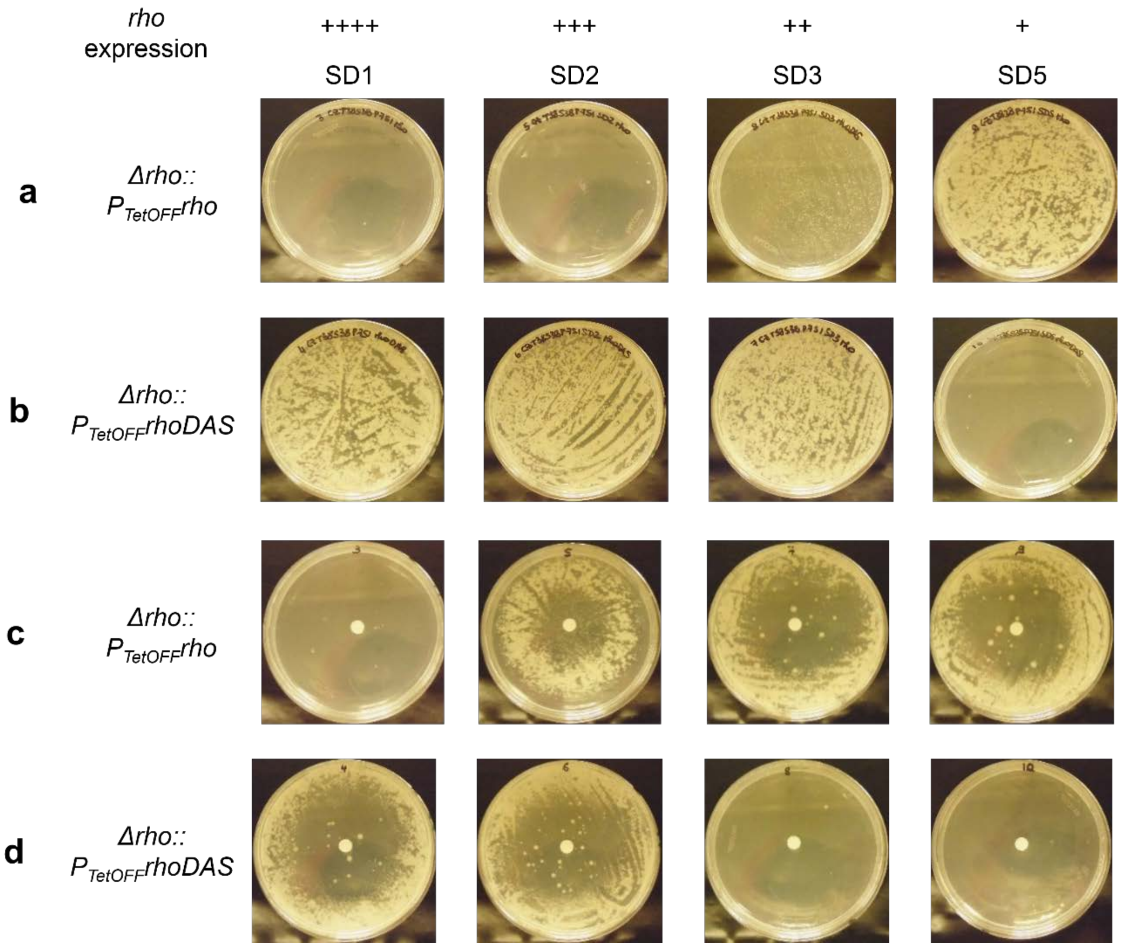

Supplementary Figure 2 │ DAS-tagging reduces the activity of Rho in *M. tuberculosis* .

The effect of the DAS+4-tag on Rho activity was evaluated by complementing the genetic inactivation of rho with different expression cassettes. Expression of Rho was varied by using translation initiation signals of different strengths (SD1>SD2>SD3>SD5) as described[1]. The constitutively expressed wt copy of *rho* of the *rho att* site mutant Δ*rho*::P_{rho}*rho* was replaced by constructs that allowed high-to-low expression of *rho* or *rho-DAS* (from high to low expression: PtetOFF-SD#1, PtetOFF-SD#2, PtetOFF-SD#3, PtetOFF-SD#5). Panels (a) and (b) show plates without atc; panels (c) and (d) show plates to which 100 ng of atc was added using a paper disc.

Strong expression of WT Rho was toxic as exemplified by the absence of growth on solid media without atc (panels a & c). The use of weaker translational initiation signals restored growth, suggesting that a moderate expression of WT Rho was sufficient to support growth. However, when using Rho-DAS+4, growth only occurred when Rho-DAS was expressed using strong translational initiation signals (SD1 to SD3) (panels b). This suggested that addition of the DAS+4-tag reduced the *in vivo* activity of Rho and caused a need for stronger expression to achieve growth.

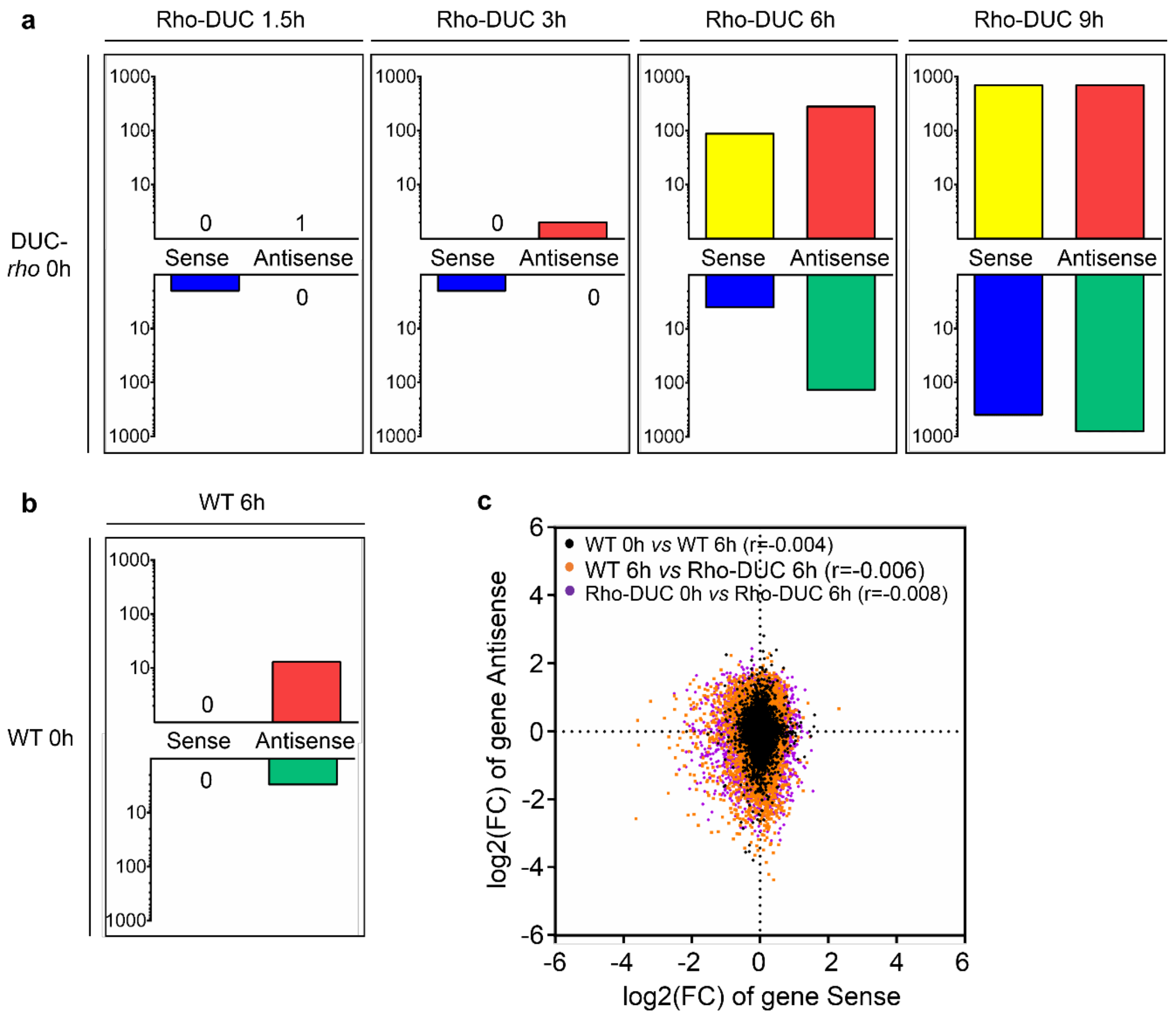

Supplementary Figure 3 │ Number of transcripts that changed in abundance after atc exposure

Transcripts were scored as changed if their abundance increased or decreased more than 2.5-fold with a False Discovery Rate [FDR] of less than 0.05. Changes are reported separately for sense and the antisense transcripts. The top histograms indicate upregulated transcripts; bottom histograms indicate downregulated transcripts.

(**a**) Gene expression changes in the Rho-DUC mutant upon *rho* silencing.

(**b**) Gene expression changes in WT after cultivation with atc.

(**c**) Correlation of the abundance changes of the antisense and sense transcripts of each gene when comparing WT 0h / WT 6h (r= -0.004), WT 6h / Rho-DUC 6h (r=-0.006) and Rho-DUC 0h / Rho-DUC 6h (r=-0.008).

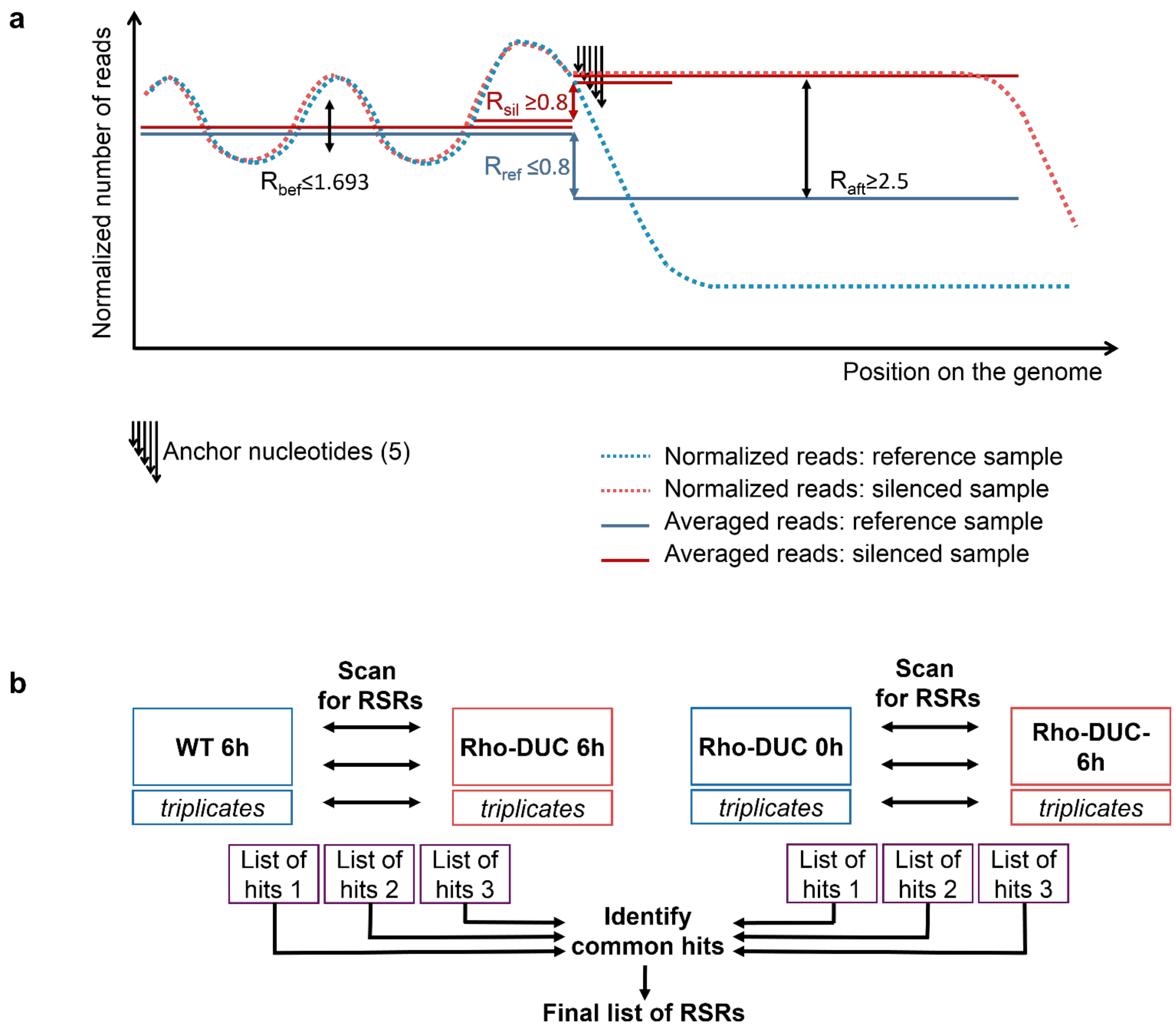

Supplementary Figure 4 | Identification of RSRs

(**a**) Schematic representation of the calculations and filters applied to define candidate RSRs. The red dashed lines represent the normalized reads of the sample in which *rho* expression was silenced; normalized reads of the reference sample are shown in blue. The small arrows represent consecutive anchor nucleotides for which the ratio between silenced and reference conditions does not decrease. To avoid the noise associated with low read numbers we excluded anchor nucleotides whose average read numbers were in the lowest quartile of the silenced sample. The 3'-end of an initial RSR hit was defined as the first nucleotide at which the ratio between silenced sample and reference sample falls back to the value of the anchor nucleotides. We then defined four ratios, R_{bef}, R_{aft}, R_{ref}, and R_{sil} as follows: (1) To

calculate R_{bef} we first defined the relevant local upstream element as the region that is equal to the length of the initial RSR hit and located right upstream of the anchor nucleotides. We then divided the average upstream reads of the silenced sample by the average upstream reads in the reference sample. (2) R_{aft} was calculated by dividing the average reads of the silenced sample after the anchor nucleotides by the corresponding average reads in the reference sample. (3) R_{ref} was defined as the ratio of average reads of the reference sample before and after the anchor nucleotides. (4) R_{sil} was calculated by dividing the average reads in the silenced sample over 250 positions after the anchor nucleotides by the average reads in the silenced sample over 250 positions before the anchor nucleotides. Candidate RSRs were then defined as regions with (i) $R_{bef} \leq 1.7$, $R_{aft} \geq 2.5$, $R_{ref} \leq 0.8$, and $R_{sil} \geq 0.8$ and for which (ii) the average reads of the reference sample after the anchor nucleotides were equal or higher than the arbitrary value of 0.0017 over the entire length of the initial RSR hit.

(**b**) Workflow for final RSR identification. Candidate RSRs identified in at least 4 of the 6 comparisons were scored as final RSRs.

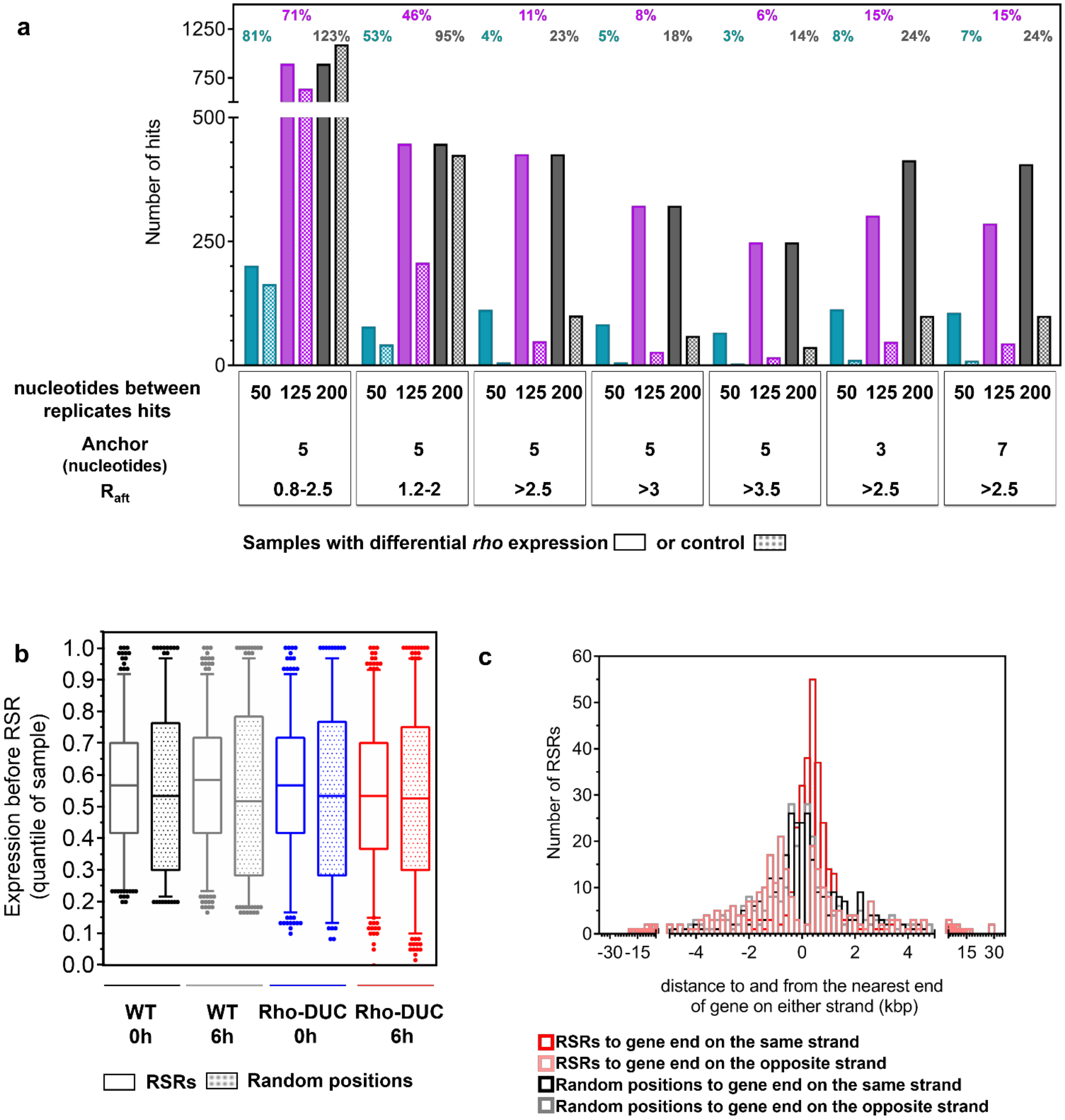

Supplementary Figure 5 | RSR identification criteria and RSR characteristics

(**a**) This figure shows how many putative RSRs are identified using different selection criteria. The parameters tested were (i) different numbers of anchor nucleotides, (ii) different values for R_{aft}, and (iii) the maximal distance allowed between hits found in different replicates to consider them as being the same RSR during cross-checking (50,125, or 200 nucleotides). The solid bars show the number of hits

obtained from the comparisons of Rho-DUC 6h with Rho-DUC 0h and WT 6h. The lighter pattern bars indicate the average number of hits obtained when either one of these comparisons was crossed with hits obtained by comparing WT 0h with Rho-DUC 0h. For each parameter change, specificity is measured with the average number of hits found in the controls expressed as a percentage of the number of hits indicated by the plain bars.

(**b**) RSRs are not biased towards regions with high or low transcript abundance. Transcript levels of the 250 bp preceding each RSR were calculated in 4 samples: WT before atc treatment (0h), WT after 6h of atc treatment, Rho-DUC before atc treatment (0h) and Rho-DUC after 6h of atc treatment. Since all samples do not have all the same overall level of RNAseq reads, the values are here expressed as quantiles part of the corresponding sample. Side by side is the same analysis of a randomly generated list of positions (boxplot shows the median value, 25 and 75 quantiles and outliers at the bottom and top 5%).

(**c**) RSRs are preferentially located around the 3'-end of the gene located upstream and on the same strand as an individual RSR. Distribution of the RSRs starts around the nearest end of a gene on the sense or antisense strand**.** Comparison of the distribution of the RSRs start positions and of a randomly generated list of positions around the nearest end of a gene on the sense or on the antisense strand.

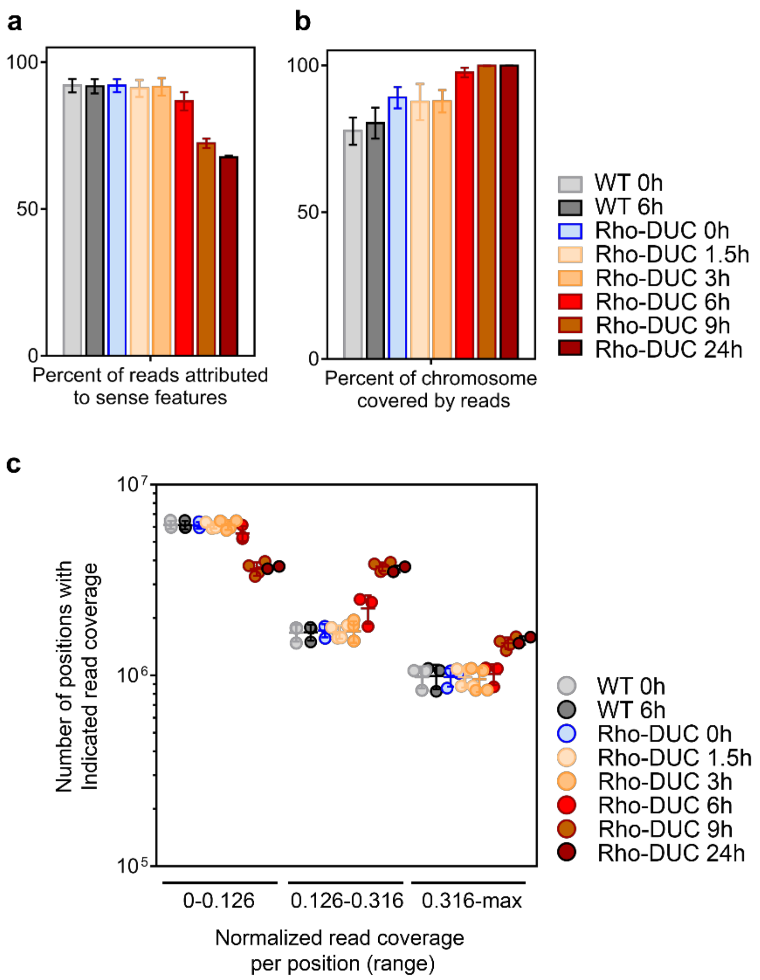

Supplementary Figure 6│ Prolonged depletion of Rho increases antisense RNA abundance and causes genome-wide pervasive transcription

(**a**) Percent of all RNAseq reads attributed to the sense direction of features in samples obtained from replicating cultures of the M. tuberculosis wt strain before or after 6h incubation with atc (wt 0h and wt 6h respectively) and the Rho-DUC strain before or after 1.5h, 3h, 6h, 9h and 24h incubation with atc (DUC 0h to 24h).

(**b**) Percent of the chromosome covered by reads in the same samples.

(**c**) Distribution of read values in the RNAseq samples. The normalized RNAseq reads from the wt and Rho-DUC strains were binned according to their values and the number of positions falling in the corresponding bin was calculated for each condition. The samples represented were prepared from the wt and the Rho-DUC *M. tuberculosis* strain before or after incubation with atc (0h and 1.5 to 24h). Color code is as in (a).

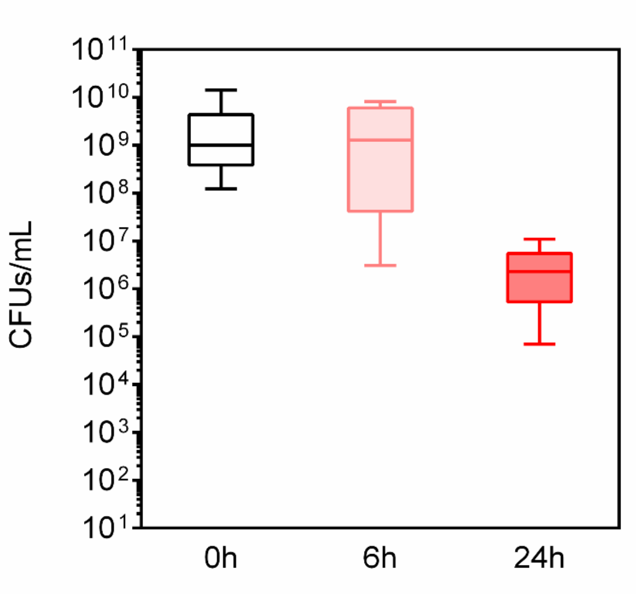

Supplementary Figure 7 | Viability of Rho-DUC decrease marginally after exposure to atc for 6h and drastically after exposure for 24h in replicating conditions

Summary of the data collected from 11 different experiments as described in figure 1c (boxplot shows the median value and 25 and 75 quantiles).

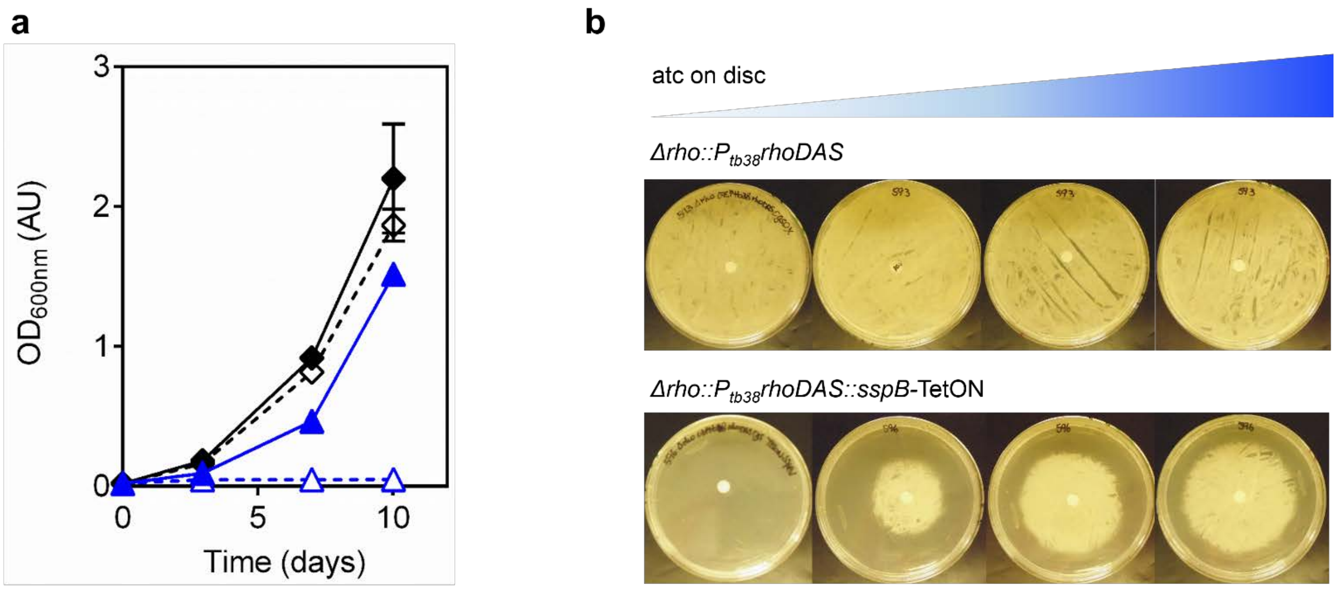

Δrho::P$_{tb38}$-rhoDAS (-) ◇ (+) ◆

Δrho::P$_{tb38}$-rhoDAS::sspB-TetON (-) △ (+) ▲

Supplementary Figure 8 | Rho-TetON mutant for conditional *rho* expression in *M. tuberculosis*

(**a**) Growth of the Rho-TetON mutant (*Δrho::Pt$_{b38}$rhoDAS::sspB*-TetON) and its parent strain (*Δrho::Pt$_{b38}$rhoDAS*) in replicating conditions in the presence or absence of 1 µg/ml of atc. Rho-DAS is expressed constitutively in Δrho::Pt$_{b38}$rhoDAS. In *Δrho::Pt$_{b38}$rhoDAS::sspB*-TetON, expression of SspB is repressed by a reverse TetR. As a consequence atc is required to prevent degradation of Rho-DAS. Data are means of triplicates cultures (±SD, some error bars too small to be seen).

(**b**) atc dependent growth of the Rho-TetON strain on solid medium. The discs contain 0, 100, 250, 500 ng of atc (left to right).

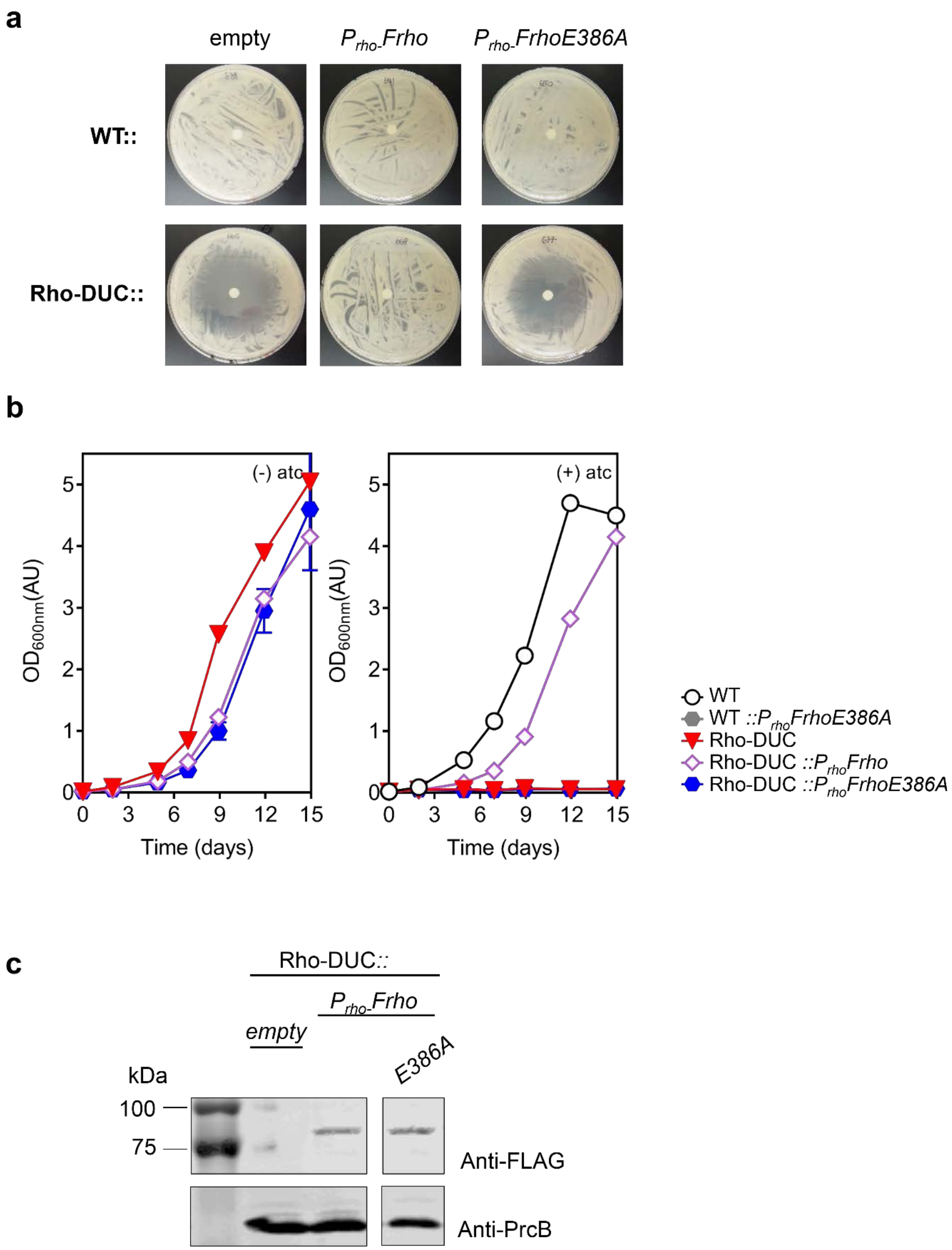
a
empty
P rho-Frho
P rho-FrhoE386A
WT::
Rho-DUC::
b
(-) atc
(+) atc
OD600nm(AU)
Time (days)
WT
WT ::P rho FrhoE386A
Rho-DUC
Rho-DUC ::P rho Frho
Rho-DUC ::P rho FrhoE386A
c
Rho-DUC::
P rho-Frho
empty
E386A
kDa
100
75
Anti-FLAG
Anti-PrcB

1

Supplementary Figure 9 │ Rho point mutant E386A fails to complement Rho Depletion in Rho-DUC

(**a**) Complementation of Rho-DUC on solid medium by a constitutively expressed FLAg-tagged copy of Rho WT (P_{rho}*rho)* or Rho point mutant E386A (P_{rho}*rhoE386A)*. 500 ng of atc was added on the discs.

(**b**) Complementation of Rho-DUC in liquid medium. The Rho-DUC strain constitutively expresses a FLAG-tagged WT Rho ($\mathrm{P_{rho}}$Frho), or a FLAG-tagged Rho-E386A (P_{rho}*FrhoE386A).* Data are means of duplicate cultures (±SD, some error bars too small to be seen).

(**c**) Western Blot of protein lysates using an anti-FLAG antibody or anti-PrcB serum (loading control) of samples grown in the absence of atc in (b). The entire blots are shown in Supplementary Figure 14.

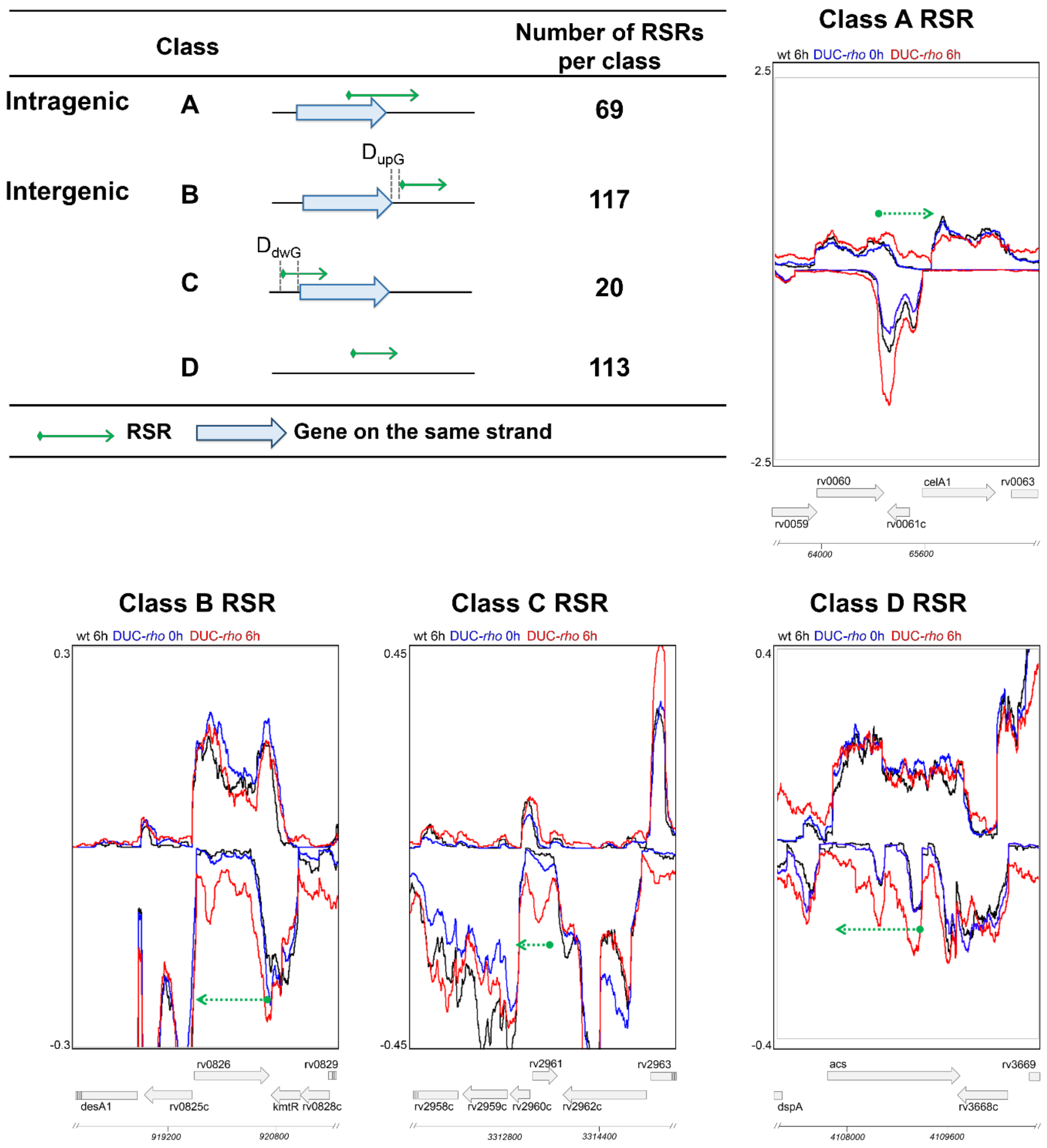

Supplementary Figure 10 | Classes of RSRs and examples

RSRs have been classified as intragenic (class A) or intergenic (class B, C, D) according to their position relative to the closest gene that is transcribed in the same direction as the RSR (DupG: Distance to upstream Gene, DdwG: Distance to downstream Gene). Some RSRs belong to two classes: intragenic RSRs reaching into another gene are counted as A and C and RSRs terminating a gene based on the D_{upG} distance and reaching into another gene are counted as B and C.

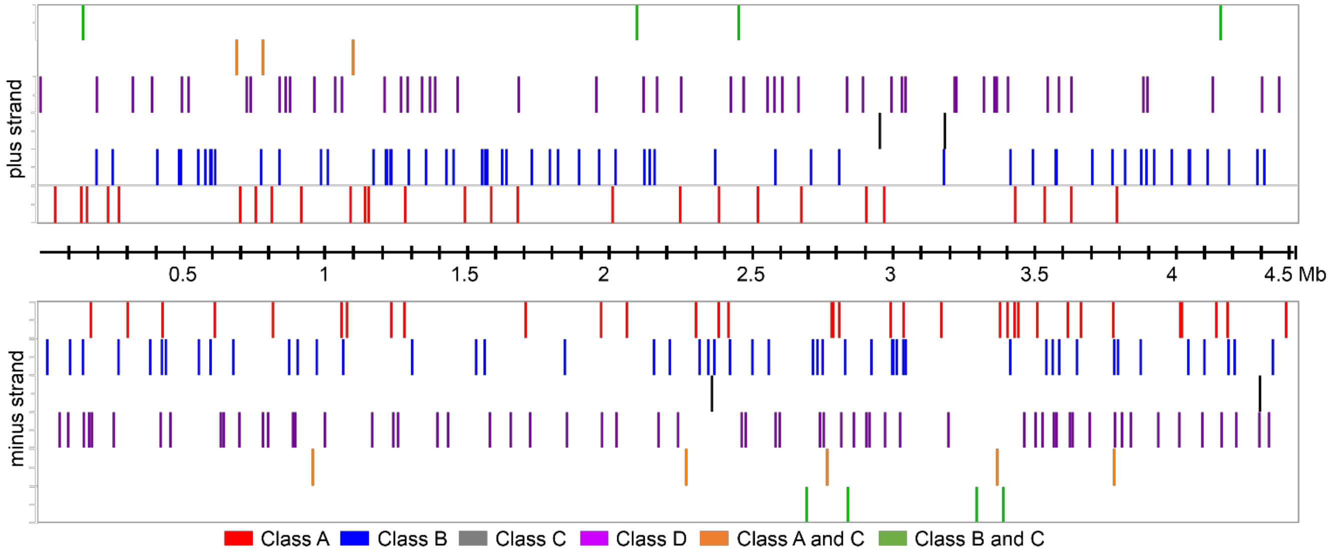

Supplementary Figure 11│ The different classes of RSRs show no clustering along the *M. tuberculosis* genome

The RSRs are represented by the position of their starts on the plus or the minus strand along the chromosome of M. tuberculosis. For each strand the RSRs are scattered on 5 levels corresponding from the middle to the exterior to the classes of RSR: A, B, C, D or both A and C and both B and C. (RSRs are represented by their start position).

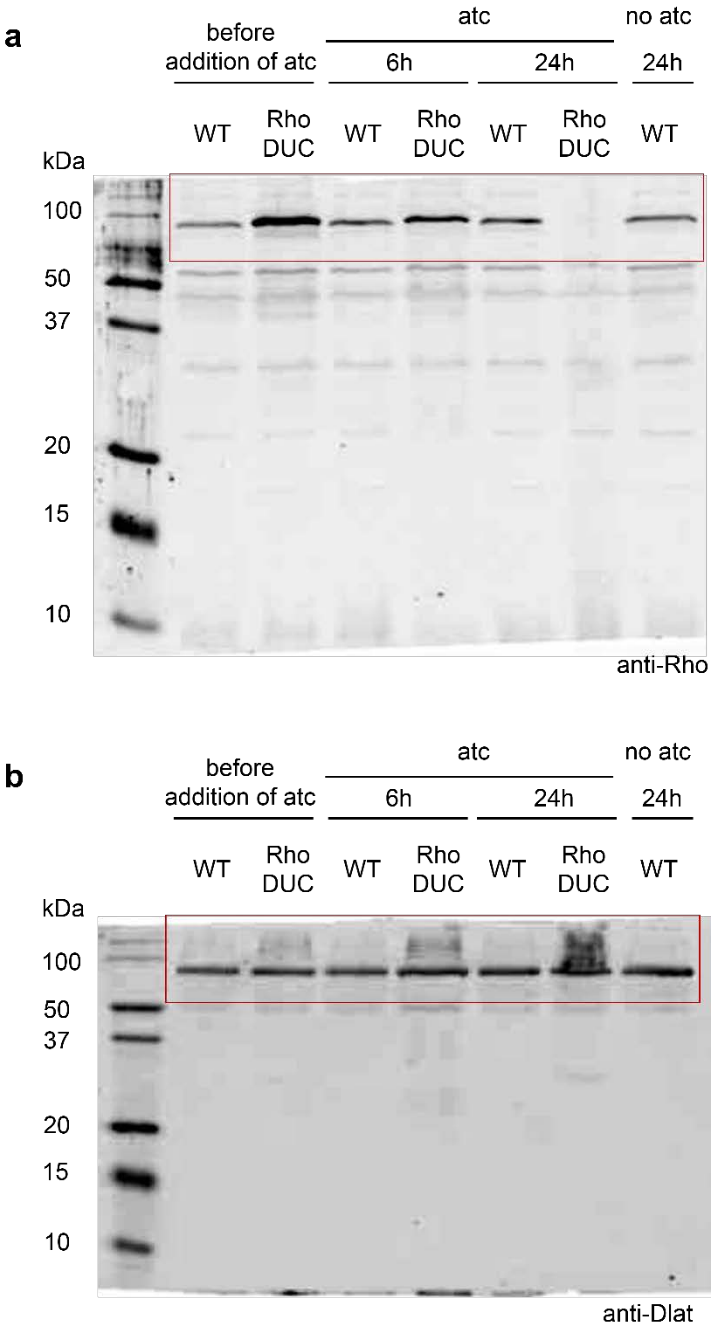

Supplementary Figure 12 | Full blots from Figure 1

Uncropped blots from Figure 1.

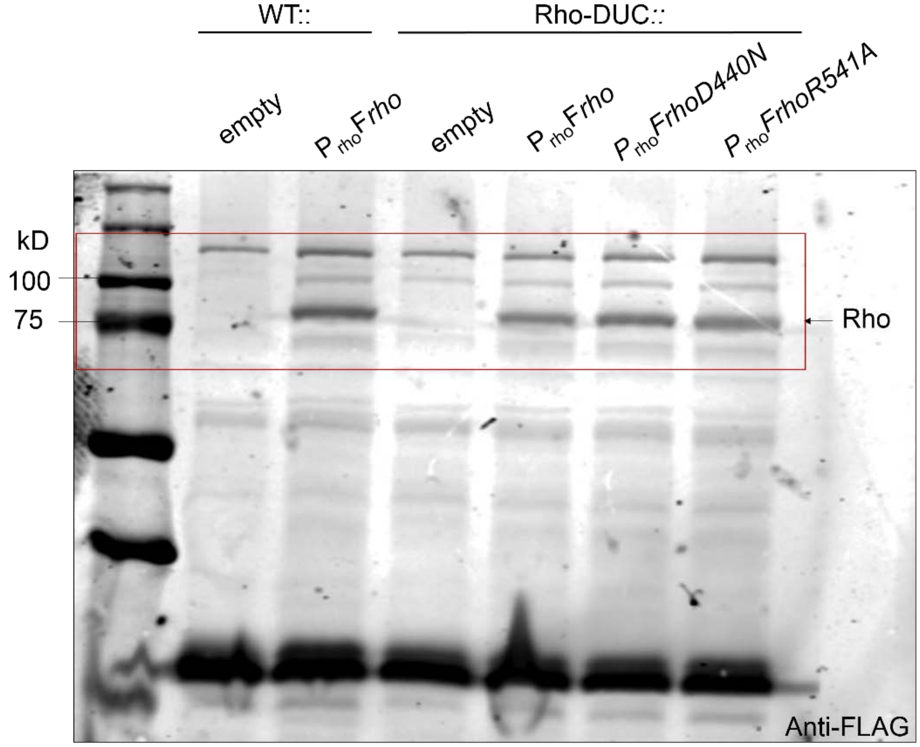

Supplementary Figure 13 | Full blot from Figure 5

Uncropped blot from Figure 5.

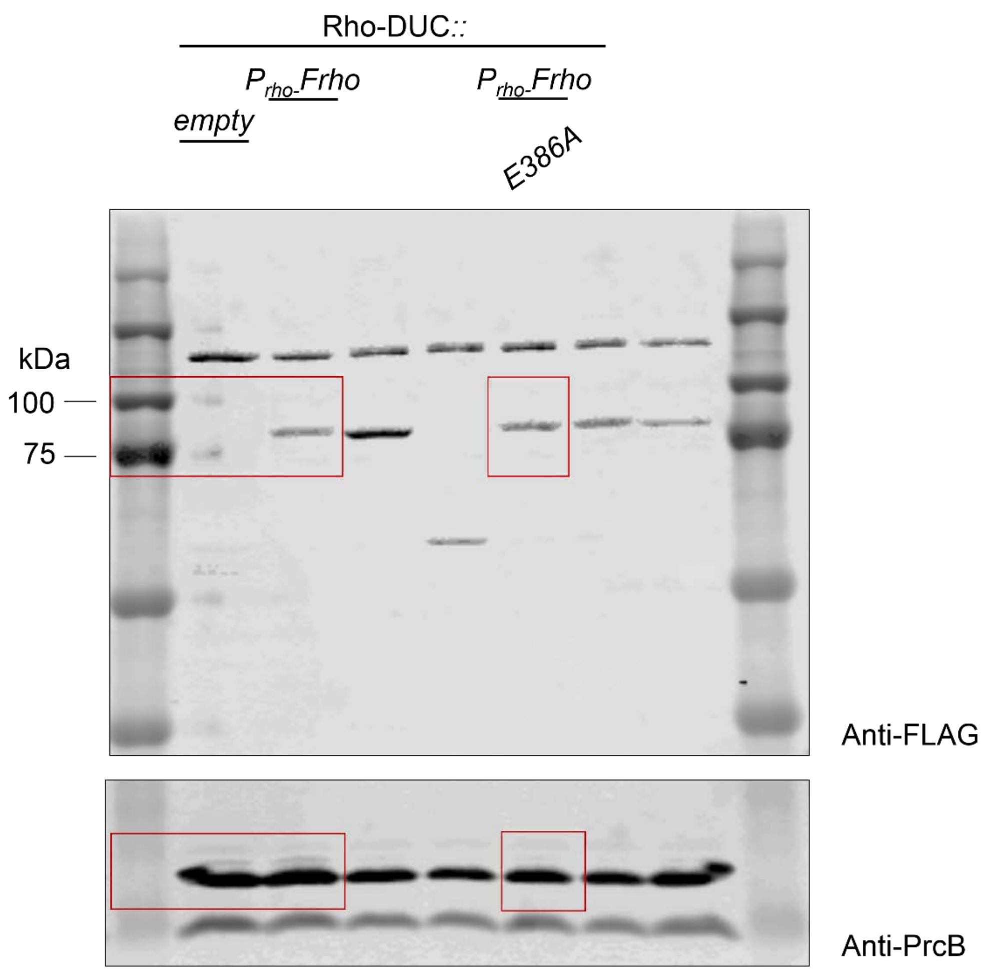

Supplementary Figure 14 | Full blots from Supplementary Figure 9

Uncropped blots from Supplementary Figure 9.

Supplementary Table 1 │ Plasmids used in this work

Plasmids	Description	origin
pNit-Rec-ET	Episomal replication, contains the enzymes for recombineering in mycobacteria	[2]
pGMCS-P_{rho}rho	Integration into the att-L5 site, contains the 600 bp upstream of the *M. tuberculosis rho* and *M. tuberculosis rho*	This work
pGMCZ-0X0X	Integration into the att-L5 site	This work
pGMCZ-P_{rho}rho	Integration into the att-L5 site, contains the 600 bp upstream of the *M. tuberculosis rho* and *M. tuberculosis rho*	This work
PGMCZ-P_{hsp60}-rhoTb	Integration into the att-L5 site, contains *M. tuberculosis* rho expressed under the control of the strong hsp60 promoter	This work
pGMCZ-T38S38-P751-rhoTb-SD1	Integration into the att-L5 site, contains *M. tuberculosis rho* expressed under the control of the promoter P751 harboring the operator *tetO4C5G,* and *revTetR*	This work
pGMCZ-T38S38-P751-rhoTb-SD2	Integration into the att-L5 site, contains *M. tuberculosis rho* expressed under the control of the promoter P751 harboring the operator *tetO4C5G,* and *revTetR*	This work
pGMCZ-T38S38-P751-rhoTb-SD3	Integration into the att-L5 site, contains *M. tuberculosis rho* expressed under the control of the promoter P751 harboring the operator *tetO4C5G,* and *revTetR*	This work
pGMCZ-T38S38-P751-rhoTb-SD5	Integration into the att-L5 site, contains *M. tuberculosis rho* expressed under the control of the promoter P751 harboring the operator *tetO4C5G,* and *revTetR*	This work
pGMCZ-T38S38-P751-rhoTb-DAS+4 SD1	Integration into the att-L5 site, contains *M. tuberculosis rho-DAS+4* expressed under the control of the promoter P751 harboring the operator *tetO4C5G,* and *revTetR*	This work
pGMCZ-T38S38-P751-rhoTb-DAS+4 SD2	Integration into the att-L5 site, contains *M. tuberculosis rho-DAS+4* expressed under the control of the promoter P751 harboring the operator *tetO4C5G,* and *revTetR*	This work
pGMCZ-T38S38-P751-rhoTb-DAS+4 SD3	Integration into the att-L5 site, contains *M. tuberculosis rho-DAS+4* expressed under the control of the promoter P751 harboring the operator *tetO4C5G,* and *revTetR*	This work
pGMCZ-T38S38-P751-rhoTb-DAS+4 SD5	Integration into the att-L5 site, contains *M. tuberculosis rho-DAS+4* expressed under the control of the promoter P751 harboring the operator *tetO4C5G,* and *revTetR*	This work

pGMCtKq29-TSC10M1-sspB-SD5	Integration into the chromosome at the attachment of the Tweety phage (att-Tweety) site, contains the gene *sspB* under the control of a promoter containing the operator *tetO*, and the gene encoding the TetR repressor *tsc10*	This work
pGMCZq1-T38S38-0X	Integration into the att-L5 site, contains *revTetR*	This work
pGMCtKq27-TSC10M1-sspB	Integration into the chromosome at the attachment of the Tweety phage (att-Tweety) site, contains the gene *sspB* under the control of a promoter containing the operator *tetO*, and the gene encoding the TetR repressor *tsc10*	This work
pGMCgS-0X	Integration into the attachment site of the giles phage (att-giles)	This work
pGMCgS-P_{rho}rhoTb	Integration into the att-giles site, contains the 600 bp upstream of the *M. tuberculosis rho* and *M. tuberculosis rho*	This work
pGMCgS-P_{rho}rhoTbD440N	Integration into the att-giles site, contains the 600 bp upstream of the *M. tuberculosis rho* and *M. tuberculosis rhoD440N*	This work
pGMCgS-P_{rho}rhoTbR541A	Integration into the att-giles site, contains the 600 bp upstream of the *M. tuberculosis rho* and *M. tuberculosis rhoR541A*	This work
pGMCgS-P_{rho}FLAGrhoTb	Integration into the att-giles site, contains the 600 bp upstream of the *M. tuberculosis rho* and N-terminal FLAG-tagged *M. tuberculosis rho*	This work
pGMCgS-P_{rho}FLAGrhoTbD440N	Integration into the att-giles site, contains the 600 bp upstream of the *M. tuberculosis rho* and N-terminal FLAG-tagged *M. tuberculosis rhoD440N*	This work
pGMCgS-P_{rho}FLAGrhoTbR541A	Integration into the att-giles site, contains the 600 bp upstream of the *M. tuberculosis rho* and N-terminal FLAG-tagged *M. tuberculosis rhoR541A*	This work
pGMCgS-P_{rho}FLAGrhoTbE386A	Integration into the att-giles site, contains the 600 bp upstream of the *M. tuberculosis rho* and N-terminal FLAG-tagged *M. tuberculosis rhoE386A*	This work
pGMCZ-P_{tb38}-rhoDAS+4	Integration into the att-L5 site, contains *M. tuberculosis* rho-DAS+4 expressed under the control of strong P_{tb38} promoter	This work
pGMCgS-T38S38-P766-7C-sspB-SD5	Integration into the att-giles site, contains the *sspB* gene under control of the promoter P766-7C harboring the operator *tetO4C5G,* and revTetR.	This work

Supplementary Table 2: *Mycobacterium tuberculosis* strains used in this work

Strains		
M. tuberculosis H37Rv	*Mycobacterium tuberculosis* H37Rv. Parent strain of all the following strains in this work.	Dr. Robert North, Trudeau Institute
Merodiploid H37Rv::CS-P_{rho}rho	*Mycobacterium tuberculosis* H37Rv expressing an additional copy of *rho*	This work
H37Rv::CZ-T38::CtK-sspB	*Mycobacterium tuberculosis* H37Rv expressing Reverse TetR from the attL5 site and sspB from the tweety integration site	This work
Δrho::P_{rho}rho	H37Rv in which rho has been deleted, expressing *rho* constitutively from the att-L5 site	This work
Δrho::PTetOFF-rhoDAS (SD1)	Derivative of Δrho::P_{rho}rho in which att-L5 site construct has been replaced by a DAS+4 tagged copy of rho transcriptionally repressed by reverse TetR in the presence of atc	This work
Δrho::PTetOFF-rhoDAS SD2	Derivative of Δrho::P_{rho}rho in which att-L5 site construct has been replaced by a DAS+4 tagged copy of rho transcriptionally repressed by reverse TetR in the presence of atc	This work
Δrho::PTetOFF-rhoDAS SD3	Derivative of Δrho::P_{rho}rho in which att-L5 site construct has been replaced by a DAS+4 tagged copy of rho transcriptionally repressed by reverse TetR in the presence of atc	This work
Δrho::PTetOFF-rhoDAS SD5	Derivative of Δrho::P_{rho}rho in which att-L5 site construct has been replaced by a DAS+4 tagged copy of rho transcriptionally repressed by reverse TetR in the presence of atc	This work
Δrho::PTetOFF-rho (SD1)	Derivative of Δrho::P_{rho}rho in which att-L5 site construct has been replaced by a copy of rho transcriptionally repressed by reverse TetR in the presence of atc	This work
Δrho::PTetOFF-rho SD2	Derivative of Δrho::P_{rho}rho in which att-L5 site construct has been replaced by a copy of rho transcriptionally repressed by reverse TetR in the presence of atc	This work
Δrho::PTetOFF-rho SD3	Derivative of Δrho::P_{rho}rho in which att-L5 site construct has been replaced by a copy of rho transcriptionally repressed by reverse TetR in the presence of atc	This work
Δrho::PTetOFF-rho SD5	Derivative of Δrho::P_{rho}rho in which att-L5 site construct has been replaced by a copy of rho transcriptionally repressed by reverse TetR in the presence of atc	This work
Rho-DUC	Derivative of Δrho::P_{rho}rho in which att-L5 site construct has been replaced by a DAS+4 tagged copy of rho transcriptionally repressed by reverse TetR in the presence of atc and expressing sspB from the tweety integration site	This work
Rho-DUC::P_{rho}rho	Rho-DUC expressing constitutively an additional copy of *rho*	This work

Rho-DUC::P_{rho}rhoD440N	Rho-DUC expressing constitutively a mutated copy of *rho*, *rhoD440N*	This work
Rho-DUC::P_{rho}rhoR541A	Rho-DUC expressing constitutively a mutated copy of *rho*, r*hoR541A*	This work
Rho-DUC::P_{rho}Frho	Rho-DUC expressing constitutively a FLAG-tagged copy of *rho*	This work
Rho-DUC::P_{rho}FrhoD440N	Rho-DUC expressing constitutively a FLAG-tagged copy of *rho*, *rhoD440N*	This work
Rho-DUC::P_{rho}FrhoR541A	Rho-DUC expressing constitutively a FLAG-tagged copy of rho, r*hoR541A*	This work
Rho-DUC::P_{rho}FrhoE386A	Rho-DUC expressing constitutively a FLAG-tagged copy of rho, r*hoE386A*	
Δrho::P_{tb38}rhoDAS+4	H37Rv in which rho has been deleted, expressing *rho* constitutively from the att-L5 site	This work
Δrho::P_{tb38}rhoDAS+4::sspB-TetON	Δrho::P_{tb38}rhoDAS+4 in which the protein SspB expressed in the absence of atc causes the degradation of Rho-DAS+4 tagged protein	This work
Δrho::P_{TetOFF}rho		

Supplementary references

1 Woong Park, S. *et al.* Evaluating the sensitivity of Mycobacterium tuberculosis to biotin deprivation using regulated gene expression. *PLoS Pathog* **7**, e1002264, doi:10.1371/journal.ppat.1002264 (2011).

2 Murphy, K. C., Papavinasasundaram, K. & Sassetti, C. M. Mycobacterial recombineering. *Methods Mol Biol* **1285**, 177-199, doi:10.1007/978-1-4939-2450-9_10 (2015).
